# Determinants of disclosure and non-disclosure of HIV-positive status, by pregnant women in rural South Africa

**DOI:** 10.1080/17290376.2018.1529613

**Published:** 2018-10-16

**Authors:** Shandir Ramlagan, Gladys Matseke, Violeta J. Rodriguez, Deborah L. Jones, Karl Peltzer, Robert A.C. Ruiter, Sibusiso Sifunda

**Affiliations:** a HIV/Aids, STI and TB Research Programme, Human Sciences Research Council, Pretoria, South Africa; b Department of Work & Social Psychology, Maastricht University, Maastricht, the Netherlands; c Research & Innovation Chief-Directorate, The National School of Government, Pretoria, South Africa; d Psychiatry and Behavioral Sciences, University of Miami Miller School of Medicine, Miami, FL, USA; e Department of Psychology, University of Georgia, Athens, GA, USA; f Department of Research & Innovation, University of Limpopo, Sovenga, South Africa

**Keywords:** Disclosure, HIV, AIDS, Woman, Pregnancy

## Abstract

Disclosure of HIV status remains one of the major challenges to the effectiveness of the prevention of mother to child transmission of HIV in rural areas in South Africa. This study aimed at assessing the determinants of HIV status disclosure among HIV infected pregnant women who have disclosed their HIV status to someone, as well as among those who have disclosed to their partners. Cross-sectional data was collected from 673 HIV sero-positive pregnant women receiving antenatal care services at 12 Community Health Centers in Mpumalanga province. Results indicated that over two-thirds (72.1%) disclosed their status to someone, while just over half (58.4%) disclosed to their partners. Multivariate analysis showed that both disclosure of ones HIV status to someone and to their male partners was significantly associated with increase in antiretroviral therapy (ART) adherence, the known HIV positive status of their partner, and male involvement during pregnancy. Participants who were diagnosed HIV positive during this current pregnancy were less likely to disclose their HIV status to someone. Non-disclosure during current pregnancy highlights a need for interventions that will encourage disclosure among HIV positive women, with a particular focus on those who are newly diagnosed. The findings also need to integrate male partner involvement and partner disclosure during pregnancy.

## ABSTRAIT

La divulgation du statut sérologique reste l’un des principaux obstacles à l’efficacité de la prévention de la transmission du VIH de la mère à l’enfant dans les zones rurales d’Afrique du Sud. Cette étude visait à évaluer les déterminants de la divulgation du statut VIH chez les femmes enceintes infectées par le VIH qui ont révélé leur statut VIH à quelqu’un, ainsi que parmi ceux qui ont divulgué à leurs partenaires. Des données transversales ont été recueillies auprès de 673 femmes enceintes séropositives pour le VIH recevant des soins prénatals dans 12 centres de santé communautaire de la province de Mpumalanga. Les résultats indiquaient que plus des deux tiers (72,1%) révélaient leur statut à quelqu’un, alors qu’un peu plus de la moitié (58,4%) divulguaient à leur partenaire. L’analyse multivariée a montré que la révélation de son statut VIH à quelqu’un et à ses partenaires masculins était significativement associée à une augmentation de l’adhésion au traitement antirétroviral (ARV), au statut séropositif connu de leur partenaire et à la participation masculine pendant la grossesse. Les participants qui ont été diagnostiqués séropositifs pendant cette grossesse étaient moins susceptibles de révéler leur statut sérologique à quelqu’un. La non-divulgation au cours de la grossesse actuelle souligne le besoin d’interventions qui encourageront la divulgation chez les femmes séropositives, en mettant particulièrement l’accent sur les personnes nouvellement diagnostiquées. Les résultats doivent également intégrer l’implication des partenaires masculins et la divulgation des partenaires pendant la grossesse.

## 1. Introduction

Disclosure of HIV serostatus has been emphasised as a crucial goal in HIV testing and counselling as well as prevention of mother to child transmission of HIV [PMTCT] protocols (Alemayehu et al., 2015; Medley, Garcia-Moreno, McGill, & Maman, 2004; UNAIDS, 2011; WHO, 2012). An individual may disclose to family members, co-workers or friends for social and emotional support or sexual partners for HIV prevention as well as support. It is crucial for pregnant women to disclose to sexual partners for them to adopt safer sexual behaviours, to prevent re-infection if they are both HIV infected, or avoid infecting the HIV negative partner (Shiyoleni, 2013). In addition, HIV disclosure to sexual partners minimise the risk of HIV transmission to the unborn baby during pregnancy, at delivery and during breastfeeding after birth (Shiyoleni, 2013). Disclosure by HIV positive women can encourage their partners to make informed reproductive health choices (Shiyoleni, 2013).

Mathematical models have shown that serostatus disclosure may contribute to the reduction of HIV transmission by 41% (Pinkerton & Galletly, 2007). The percentage of women who disclose their status to at least one person varies widely across regions. Prevalence rates of HIV serostatus disclosure to any person ranged from 5.0% to 96.7% (Tam, Amzel, & Phelps, 2015; Kiula, Damian, & Msuya, 2013; Patel et al., 2012). Among pregnant women prevalence of HIV serostatus disclosure to sexual partners ranged from 16.7% to 78% (Gaillard et al., 2002; Kilewo et al., 2001; Brou et al., 2007; Nebié et al., 2001; Desgrées-Du-Loû et al., 2009; Kiula et al., 2013). In a South African study among HIV positive new mothers in Nkangala district, 79% reported that they disclosed their status to someone and 65% disclosed to their partners (Peltzer & Mlambo, 2013). A study conducted in Zimbabwe reported that 78% of women had disclosed their status to their current partner and the majority (85–98%) experienced a positive reaction (Patel et al., 2012). In contrast, a study conducted in Sub-Saharan Africa reported much lower rates of disclosure to partner, at 37%, among HIV infected pregnant women (Hardon et al., 2012).

Disclosure of their HIV status by women to their male partner was found to significantly improve antiretroviral adherence during pregnancy in Tanzania (Kirsten et al., 2011) and Nigeria (Ekama et al., 2012). There were also significant increases to adherence to the PMTCT protocol in the post-natal period, such as returning for post counselling, increased adherence to ART medication, and increased condom use (Ramirez-Ferrero & Lusti-Narasimhan, 2012; Patel et al., 2012), as well as lower levels of depression (Patel et al., 2012). It is possible that these reported improvements in adherence as a result of HIV serostatus disclosure may, in part, be due to increased male involvement and support. Increasing male partner involvement (MPI) in PMTCT programmes can help women and their partners to understand and cope with the diagnosis (Albrecht et al., 2006; Maru et al., 2009; Sagay et al., 2006). In South Africa, disclosure and male partner involvement have similarly been shown to improve antiretroviral adherence during pregnancy among HIV positive women (Peltzer, Sikwane, & Majaja, 2011).

Despite several benefits associated with HIV serostatus disclosure, there are various barriers that prevent women from disclosing to their sexual partners. In South Africa, significant progress has been achieved in the implementation of PMTCT programmes. However, these improvements have, for the most part, occurred in urbanised areas, with rural areas remaining at unacceptably high levels of MTCT of HIV (Wettstein et al., 2012). Lack of HIV status disclosure has been identified as one of the major barriers to PMTCT effectiveness in rural areas (Bajunirwe & Muzoora, 2005; Bancheno, Mwanyumba, & Mareverwa, 2010; Mepham, Zondi, Mbuyazi, Mkhwanazi, & Newell, 2011; Tabana et al., 2012) as well as posing as a challenge to implementation of PMTCT programmes in overburdened clinics (Hardon et al., 2012; Kuonza, Tint, Harris, & Nabukenya, 2011; Turan et al., 2011, 2012).

Studies among HIV infected women have shown that HIV disclosure could lead to undesirable outcomes such as intimate partner violence (Colombini et al., 2016; Medley et al., 2004; Shamu et al., 2014), stigma and discrimination (French, Greeff, & Watson, 2015). Being diagnosed HIV positive has also been shown to cause post-traumatic stress (Olley, Zeier, Seedat, & Stein, 2005). A review of 17 studies (15 in Sub Saharan Africa and 2 from South East Asia) showed that between 3.5% and 14.6% of women reported experiencing a violent reaction from a partner following disclosure (Medley et al., 2004).

A review of factors associated with HIV serostatus disclosure in Sub-Saharan Africa (Tam et al., 2015) identified four main determinants, i.e. factors related to the woman herself (younger age, first pregnancies, knowing someone with HIV, lower levels of internalised stigma, and lower levels of avoidant coping), the partner (prior history of HIV testing and higher levels of educational attainment), their partnership (no history of domestic violence and financial independence), and the household (higher quality of housing and residing without spouses or extended family members). On the contrary, other studies have shown low rates of disclosure among younger women compared to older women (Kadowa & Nuwaha, 2009; Ssali et al., 2010).

In a South African study among HIV positive pregnant women, being married, prior discussion about testing, having a partner with tertiary education and less experience of violence were identified as factors associated with having disclosed to partners (Makin et al., 2008). Peltzer and Mlambo (2013) found that among HIV positive new mothers in South Africa, having been diagnosed HIV positive more than two years ago and knowing their partner is HIV positive among other factors, increased the likelihood of disclosure. Better housing, low financial dependence on partners, and knowing someone with HIV were associated with disclosure to any other person (Makin et al., 2008). In Tanzania, HIV-disclosure to partners was more likely among pregnant women who were under 25 years old, who knew their HIV status before the current pregnancy, and discussed with their partner before testing (Kiula et al., 2013). Dependency on the partner for food/rent/school fees, led to lower odds of disclosure to partners (Kiula et al., 2013).

This study examines the socio-demographic, HIV related, and psychosocial determinants of HIV status disclosure and non-disclosure among HIV sero-positive pregnant women in rural South Africa.

## 2. Methodology

### 2.1. Design

The methodology and data presented here forms part of the Protect Your Family (PYF) randomised controlled trial described in detail elsewhere (Jones et al., 2014). The study was conducted in Mpumalanga province, South Africa. Within the province, 12 community health centres (CHC) from the Nkangala District Municipality and Gert Sibande District Municipality were selected as study sites. As per the study protocol,

twelve CHCs were matched in a 1:1 ratio according to patient census and average ANC volume, and one clinic in each pair was randomly assigned to the experimental or control condition using a computer program written by the data manager. The matched clinics were then assigned to the opposite condition. (Jones et al., 2014, p.415)

Over a 12 month period, from April 2014 to March 2015, cross-sectional data were gathered.

### 2.2. Sample and procedure

A total of 673 women were recruited by trained PYF staff and enrolled into this study. In order to be enrolled, women had to be aged 18 years or older, less than six months pregnant, HIV-seropositive, and having a current partner. Gestational age was determined by CHC nurses utilising anti-natal protocols and HIV seropositive status was determined utilising South African Department of Health PMTCT guidelines (Department of Health, 2014). It was not necessary for the current partner to be the father of the unborn baby. Once a potential respondent was identified, they were introduced to the study information sheet by the trained PYF staff member, in the language of their preference including English, isiZulu, or seSotho. The study information sheet was read out to them while they followed on their own copy. Respondents were given an opportunity to ask questions after each paragraph. The information sheet and consent form ensured participants knew that confidentiality was of the highest priority in this study and their HIV status and data they provided could not be linked to them personally. Upon agreeing to participate, they were asked to provide written informed consent.

Briefly, enrolled participants then completed the assessment in their above mentioned preferred language utilising the Questionnaire Development System v3.0 (QDS^TM^) Audio Computer-Assisted Self-Interview (ACASI) software (NIMH Multisite HIV/STD Prevention Trial for African American Couples Group, 2008). All participants were provided with over-ear headphones and given brief training by the PYF staff member on the usage of the laptop touchscreen, headphones, and ACASI software. Once the assessment was completed, the PYF staff member saved the data onto the touch screen laptop utilising a unique study participant identification number as well as date when the interview occurred. Participants were then thanked for their time and compensated with R50.00 (South African Rand = US$4.72). The data were backed up onto an external memory drive at the end of every day by the PYF staff member at each CHC. Once every week, the data were collected in person from each CHC by a PYF study coordinator and transferred to the study head office in Pretoria and handed over to the study statistician.

### 2.3. Ethical approval

Ethical approval was granted by the Human Sciences Research Council (HSRC) Research Ethics Committee (REC), protocol approval number REC4/21/08/13 as well as the University of Miami, Miller School of Medicine, Institutional Review Board (IRB ID: 20130238 (CR00006122). As the entire study was being conducted within Mpumalanga Province, South Africa, study approval was also obtained from the Department of Health and Welfare, Mpumalanga Provincial Government, South Africa.

### 2.4. Measures

#### 2.4.1. Primary outcome measure

Disclosure of HIV status which is the primary outcome was derived from two independent questions regarding the disclosure of the participants HIV positive sero-status. The first outcome question was ‘have you disclosed your HIV status to anyone (someone),’ with response options Yes or No. This question also included disclosure to one partner. The second outcome question was ‘have you disclosed your HIV status to your partner,’ with response options Yes vs No. This study defined a male partner as either the women’s husband, or the current baby’s father, or the women’s current male sexual partner, or the women’s trusted male friend who was actively involved in her life (Jones et al., 2014).

#### 2.4.2. Explanatory measures

Socio-demographic items were assessed with questions including age, language (Nguni vs others), education (completed Grade 11 or less and completed Grade 12 or more), relationship status (living together with current partner or not), employment status (not employed versus employed, volunteer or student), income per month in South African Rand (ZAR) where the South African Government child grant was R310.00 (UD$29.27) during the study period; number of children (none vs one or more); planned or unplanned pregnancy; and alcohol use of more than 2 drinks on at least on one occasion in the past 4 weeks (yes vs no).

HIV related measures included being diagnosed with HIV during this pregnancy (yes vs no); if you have a living child, are any of your children HIV positive (yes vs no); and is your current partner HIV positive (yes vs no). An adapted version of the Visual Analog Scale (VAS; Giordano, Guzman, Clark, Charlebois, & Bangsberg, 2004) was utilised to measure adherence to antiretroviral therapy (ART). Adherence was noted as 100% if all ART doses were taken over the previous seven days. Range was from 0% (not taken on any single day) to 100% (7 days). Stigma was assessed using the nine-item AIDS-Related Stigma Scale (ARSS; Kalichman et al., 2005). Scores on the scale range from 0 to 8, where higher scores indicate greater levels of stigma. Cronbach’s Alpha reliability coefficient for the scale in this study was *α* = .73.

Intimate partner violence (IPV) was assessed using an adapted 18-item version of the Conflict Tactics Scale 18 (CTS-18; Straus, 1979). The scale asked to rate the number of times she and her partner may have engaged in the conflict in the past month on a seven point scale of 0 (*Never*) to 6 (*More than 20 times*). A higher score, is indicative of increased IPV. Cronbach’s Alpha reliability coefficient for the scale in this study was *α* = .84. Mental status was assessed using the 10-item Edinburgh Postnatal Depression Scale 10 (EPDS-10; Cox, Holden, & Sagovsky, 1987). Scores range from 0 to 30, where the higher the score, the more the likelihood of depression being experienced. This paper utilised the validated cut-off score for South African populations at score 12 (Lawrie, Hofmeyr, De Jager, & Berk, 1998). Cronbach’s Alpha reliability coefficient for the scale in this study was α=.66. Male involvement was assessed using a Yes/No item to enquire whether or not the respondent’s male partner knew what occurred during in the antenatal clinic visit.

### 2.5. Data analysis

Summary statistics (means, frequencies and percentages) were used to describe the study sample utilising SPSS version 24.0 for Windows (IBM Corp, 2013). Cronbach’s Alpha reliability coefficients were calculated for scales used in the analyses, including IPV, ARSS, and EPDS scales. Bivariate analyses and multivariate logistic regressions were used to investigate associations between the primary outcome, disclosure of HIV status, and socioeconomic, HIV related, and behavioural variables. Associations were considered significant at *P* < .05. All statistically significant variables in the bivariate analyses were included in the multivariate model.

## 3. Results

### 3.1. Sample characteristics and HIV disclosure

The 673 HIV positive pregnant women in this study had a mean age of 28.39 years (SD = 5.73), with the majority (58%) between the ages of 18–29 years old. Over two-thirds (72.1%) disclosed their status to someone, while just over half (58.4%) disclosed to their partners. All respondents indicated they were in a relationship with a partner, however a high proportion of about 62% were not living together with their partner. Among those respondents who were not living together with their partner, 47% did not disclose their HIV positive status to their partner and among those living with their partners, 34% did not disclose their HIV positive status to their partner they are living with. About 78% reported that they were unemployed and a third reported earning a monthly income of less than R310. Of the respondents that earned less than R310 per month, 33% did not disclose their HIV positive status to someone and 44% did not disclose their status to their partners (see Table 1). A fifth (21%) of respondents reported that they had no children and of these, 63% disclosed their HIV status to someone and half (50%) disclosed to their partner.

**Table 1.** Sample characteristics and HIV disclosure.

Sociodemographics	Sample	Disclosed HIV status to Someone		Disclosed HIV status to Partner	
		Yes	No	Yes	No
	*N*(%)	*n*(%)	*n*(%)	*n*(%)	*n*(%)
All	673(100)	485(72.1)	188(27.9)	393(58.4)	280(41.6)
Language					
Nguni languages	546(81.1)	397(72.7)	149(27.3)	318(58.2)	228(41.8)
Other languages	127(18.9)	88(69.3)	39(30.7)	75(59.1)	52(40.9)
Educational attainment					
Grade 12 and more	192(28.5)	141(73.4)	51(26.6)	117(60.9)	75(39.1)
Grade 11 and less	481(71.5)	344(71.5)	137(28.5)	276(57.4)	205(42.6)
Relationship status					
Not living together	419(62.3)	299(71.4)	120(28.6)	224(53.5)	195(46.5)
Living together	254(37.7)	186(73.2)	68(26.8)	169(66.5)	85(33.5)
Employment status					
Not employed	527(78.3)	379(71.9)	148(28.1)	314(59.6)	213(40.4)
Employed, Volunteer or Student	146(21.7)	106(72.6)	40(27.4)	79(54.1)	67(45.9)
Income (ZAR)per month					
<R310 (child grant)	221(32.8)	148(67.0)	73(33.0)	123(55.7)	98(44.3)
R311 or more	452(67.2)	337(74.6)	115(25.4)	270(59.7)	182(40.3)
Number of living children					
None	139(20.7)	88(63.3)	51(36.7)	70(50.4)	69(49.6)
One or more	534(79.3)	397(74.3)	137(25.7)	323(60.5)	211(39.5)
Unplanned pregnancy					
No	317(47.1)	226(71.3)	91(28.7)	184(58.0)	133(42.0)
Yes	356(52.9)	259(72.8)	97(27.2)	209(58.7)	147(41.3)
Alcohol use of more than 2 drinks on at least on one occasion in the past 4 weeks					
No	581(86.3)	419(72.1)	162(27.9)	341(58.7)	240(41.3)
Yes	92(13.7)	66(71.7)	26(28.3)	52(56.5)	40(43.5)

### 3.2. Health and behaviour characteristics and HIV disclosure

In the study sample, just over half (54%) of the respondents were diagnosed with HIV during the current pregnancy (see Table 2 below). Of these respondents, almost two thirds (63%) disclosed their HIV status to someone yet only about half (49%) disclosed their HIV status to their partner. Table 2 shows that well over two thirds (69%) of HIV positive pregnant women were 100% adherent to their ART medication and of these adherent respondents, a high three quarters (76%) disclosed their status to any one and just over three fifths (62%) disclosed their HIV positive status to their partners. A quarter (25%) of respondents reported that their partner was diagnosed as HIV positive and of these, a very high 87% disclosed their HIV status to their partner (see Table 2). Only 58% of respondents stated that their partner is involved in their pregnancy as their partner knows what takes place during the ante-natal care clinic, and of these respondents, 68% declared their HIV positive status to their partner.

**Table 2.** Health and behaviour characteristics and HIV disclosure.

	Sample	Disclosed HIV status to Someone		Disclosed HIV status to Partner	
		Yes	No	Yes	No
	*N*(%)	*n*(%)	*n*(%)	*n*(%)	*n*(%)
All	673(100)	485(72.1)	188(27.9)	393(58.4)	280(41.6)
Diagnosed with HIV in this pregnancy					
No, before	308(45.8)	255(82.8)	53(17.2)	214(69.5)	94(30.5)
Yes	365(54.2)	230(63.0)	135(37.0)	179(49.0)	186(51.0)
Has HIV positive children					
No or do not know	506(94.8)	134(26.5)	372(73.5)	303(59.9)	203(40.1)
Yes	28(5.2)	3(10.7)	25(89.3)	20(71.4)	8(28.6)
*VAS adherence					
non adherent	210(31.2)	135(64.3)	75(35.7)	104(49.5)	106(50.5)
100% adherent	463(68.8)	350(75.6)	113(24.4)	289(62.4)	174(37.6)
HIV positive partner					
No or do not know	506(75.2)	336(66.4)	170(33.6)	248(49.0)	258(51.0)
Yes	167(24.8)	149(89.2)	18(10.8)	145(86.8)	22(13.2)
Intimate partner violence					
No mild or No severe physical violence	541(80.4)	148(27.4)	393(72.6)	324(59.9)	217(40.1)
Mild or severe physical violence	132(19.6)	40(30.3)	92(69.7)	69(52.3)	63(47.7)
Stigma					
No (Score = 0)	400(59.4)	114(28.5)	286(71.5)	231(57.8)	169(42.3)
Yes (Score = 1–8)	273(40.6)	74(27.1)	199(72.9)	162(59.3)	111(40.7)
Depression					
EDS score of 0–12	345(51.3)	257(74.5)	88(25.5)	213(61.7)	132(38.3)
EDS score of 13 and more	328(48.7)	228(69.5)	100(30.5)	180(54.9)	148(45.1)
Male involvement					
No	281(41.8)	181(64.4)	100(35.6)	125(44.5)	156(55.5)
Yes	392(58.2)	304(77.6)	88(22.4)	268(68.4)	124(31.6)

*VAS = Visual Analogue Scale.

### 3.3. Association of demographic, socioeconomic, health and behaviour characteristics to disclosure of HIV status

In Table 3, the results from the univariate logistic regression indicated that older age, increased ART adherence, having an HIV positive partner, and male partner involvement, were positively associated with HIV status disclosure to someone and to partners. Additionally, having a child/children, and being diagnosed with HIV during the current pregnancy, were negatively associated with HIV status disclosure to someone and to partners. Furthermore, Table 3 goes on to show that income and relationship status were associated with HIV status disclosure to someone and HIV status disclosure to partner, respectively. Participants with an income of R311 and more per month were less likely to disclose their HIV-positive status to someone and those respondents who are living together with their partner were more likely to disclose their HIV-positive status to their partners.

**Table 3.** Association of demographic, socioeconomic, health and behaviour characteristics to disclosure of HIV status.

	Disclosed HIV status to Someone		Disclosed HIV status to Partner	
	Cr OR (95% CI) *p	Adj OR (95% CI) *p	Cr OR (95% CI) *p	Adj OR (95% CI) *p
Age (scale)	1.04(1.01–1.07) *p = 0.022	1.01(0.97–1.04) *p* = 0.770	1.04(1.01–1.07) *p = 0.004	1.02(0.99–1.06) *p* = 0.168
Language				
Nguni languages	0.85(0.56–1.29) *p* = 0.440		1.03(0.70–1.53) *p* = 0.867	
Other languages (ref)				
Educational attainment				
Grade 12 or more	1.10(0.76–1.60) *p* = 0.616		1.16(0.82–1.63) *p* = 0.398	
Grade 11 and less (ref)				
Relationship status				
Not living together	1.10(0.77–1.56) *p* = 0.601		1.73(1.25–2.39) *p = 0.001	1.31(0.91–1.88) *p* = 0.147
Living together (ref)				
Employment status				
Not employed	1.04(0.64–1.46) *p* = 0.870		0.80(0.55–1.56) *p* = 0.236	
Employed, student, volunteer (ref)				
Income (ZAR)per month				
<R310	0.69(0.49–0.98) *p = 0.040	0.76(0.51–1.12) *p* = 0.168	0.85(0.61–1.17) *p* = 0.314	
R311 or more (ref)				
Number of living children				
None	0.60(0.40–0.89) *p = 0.010	0.78(0.50–1.24) *p* = 0.295	0.66(0.46–0.96) *p = 0.032	0.95(0.61–1.46) *p* = 0.802
One or more (ref)				
Unplanned pregnancy				
No	1.08(0.77–1.51) *p* = 0.674		1.03(0.76–1.40) *p* = 0.862	
Yes (ref)				
Alcohol use of 2 or more drinks on at least on one occasion in the past 4 weeks				
No	0.98(0.62–1.60) *p* = 0.940		0.92(0.59–1.43) *p* = 0.695	
Yes (ref)				
Diagnosed with HIV during this pregnancy				
No	0.35(0.25–0.51) *p = 0.000	0.42(0.29–0.62) *p = 0.000	0.42(0.31–0.58) *p = 0.000	0.54(0.38–0.77) *p = 0.001
Yes (ref)				
Has HIV positive children				
No	3.00(0.89–10.10) *p* = 0.076		1.68(0.72–3.88) *p* = 0.228	
Yes (ref)				
VAS adherence				
non adherent	1.72(1.21–2.45) *p = 0.003	1.56(1.07–2.28) *p = 0.020	1.69(1.22–2.35) *p = 0.002	1.48(1.03–2.13) *p = 0.035
100% adherent (ref)				
HIV positive partner				
No or Don’t Know	4.19(2.48–7.06) *p = 0.000	2.94(1.71–5.06) *p = 0.000	6.86(4.24–11.09) *p = 0.000	4.99(3.03–8.21) *p = 0.000
Yes (ref)				
Intimate partner violence				
No	0.87(0.57–1.31) *p* = 0.499		0.73(0.50–1.06) *p* = 0.112	
Yes (ref)				
Stigma (Scale)	1.06(0.93–1.21) *p* = 0.393		1.07(0.95–1.20) *p* = 0.282	
Depression				
No/Low	0.78(0.56–1.09) *p* = 0.150		0.75(0.55–1.03) *p* = 0.071	
High (ref)				
Male involvement				
No	1.91(1.36–2.68) *p = 0.000	1.67(1.17–2.40) *p = 0.005	2.70(1.96–3.70) *p = 0.000	2.34(1.66–3.30) *p = 0.000
Yes				

Notes: Cr OR = crude Odds Ratio; Adj OR = Adjusted Odds Ratio.

*95% significance.

Finally, in multivariate analysis, increase in ART adherence, HIV positive partner, and male involvement, were significantly associated with both disclosure of HIV status to someone and disclosure of HIV status to partner. Participants who were 100% adherent to ART, those who had an HIV positive partner, and those whose male partner was involved in current pregnancy were more likely to disclose their HIV status to someone and to the partner (see Table 3, column Adj OR). Multivariate analysis further showed that participants who were diagnosed HIV positive during current pregnancy were less likely to disclose their HIV status to someone nor to their partner.

## 4. Discussion

This study examined the prevalence of HIV-positive status disclosure and non-disclosure among HIV positive pregnant women in rural South Africa. The results of this study highlight the high levels of overall non-disclosure, where just over two fifths of respondents did not disclose their HIV positive status to their male partners and over a quarter did not disclose to someone. A statistically significant association was found between those who were diagnosed with HIV during this current pregnancy, and both non-disclosure of their HIV-positive status to someone and non-disclosure of their HIV-positive status to their partner.

In line with PMTCT guidelines, women are initiated on ART on the same day as receiving an HIV diagnosis (DoH, 2014). Non-disclosure of HIV positive status among those newly diagnosed pregnant women has been attributed to post-traumatic stress disorder following HIV diagnosis (Olley et al., 2005). This occurs among many women testing HIV positive for the first time in pregnancy, during the antenatal period, despite counselling efforts. In this study, about half of the respondents were found to be depressed. This could be extremely traumatic as the respondents would have found out that they are pregnant and HIV positive almost around the same time they were being enrolled into the study. Most common reasons cited for non-disclosure of HIV status were not being emotionally ready to disclose, fear of negative reactions such as rejection, discrimination and violence (Visser, Neufeld, de Villiers, Makin, & Forsyth, 2008). Non-disclosure places these pregnant women and their unborn child at greater risk of HIV reinfection, increased viral load, increased levels of depression, and decreases their potential support system emanating from the stigma and discrimination experiences (Shiyoleni, 2013).

This study found that over two thirds of the HIV positive pregnant women were 100% adherent to their ART medication. A strong positive relationship was also found between adherence to ART and disclosure of HIV status to someone and disclosure to partner. This finding is consistent with previous research in Mpumalanga province, South Africa (Peltzer et al., 2011), in Tanzania (Kirsten et al., 2011), and in Nigeria (Ekama et al., 2012). Increased MPI has been shown to help provide support for women with HIV diagnosis, which in turn, increase adherence to ART (Albrecht et al., 2006; Maru et al., 2009; Peltzer et al., 2011).

In fact, this study found a strong positive relationship between male partner involvement during pregnancy and disclosure of HIV status. Evidence shows that increasing positive male partner involvement in pregnancy can help women and their partners to understand and cope with the HIV diagnosis (Albrecht et al., 2006; Maru et al., 2009; Sagay et al., 2006). In addition, women who had an HIV positive partner were more likely to disclose their HIV status to their partners as well as to others. This finding implies that the partner could have also disclosed his HIV positive status at some point. Increased ability of women to safely disclose their HIV-positive status to male partners is therefore essential for increased uptake and effectiveness of PMTCT services (Bancheno et al., 2010; Mepham et al., 2011; Tabana et al., 2012).

## 5. Limitations of the study

A cross-sectional study design was used which is limited to determining the association between an outcome and risk factors, and therefore cannot infer causality between HIV status disclosure and associated factors. In addition, only self-report measures were used in the present study, which predisposes participants to social desirability bias. Nevertheless, the findings of this study adds to existing literature.

## 6. Conclusion

The findings of this study highlight the need to encourage disclosure as a way to promote ART adherence among HIV positive pregnant women. The findings also highlight the importance of male partner involvement during pregnancy in order to encourage disclosure. In addition, more research is needed on other ways to encourage disclosure among newly diagnosed HIV positive pregnant women during initiation in the PMTCT programme. This study recommends increased psychosocial support for newly diagnosed HIV positive pregnant women in order to encourage disclosure to their male partners.
